# Polymicrobial infection in acute biliary infection: clinical impact, resistance, and risk factors

**DOI:** 10.3389/fcimb.2026.1893457

**Published:** 2026-10-23

**Authors:** Quanquan Luo, Yunfei Hua, Chengsi Zhao, Weijie Yao, Zuozheng Wang

**Affiliations:** 1 First Clinical Medical College, General Hospital of Ningxia Medical University, Yinchuan, China; 2 Department of Hepatobiliary Surgery, General Hospital of Ningxia Medical University, Yinchuan, China

**Keywords:** acute biliary infection, antimicrobial resistance, bile culture, polymicrobial infection, sepsis, SIRS

## Abstract

**Background:**

Polymicrobial infection is frequently observed in acute biliary infection; however, its clinical significance, antimicrobial resistance patterns, and associated risk factors remain incompletely understood.

**Methods:**

This retrospective study included patients with acute biliary infection who underwent endoscopic retrograde cholangiopancreatography (ERCP) or percutaneous transhepatic gallbladder drainage (PTGD) with bile culture between August 2020 and August 2023. Patients were categorized into monomicrobial and polymicrobial infection groups based on bile culture results. Clinical characteristics, microbial profiles, antimicrobial resistance patterns, and inflammatory severity were compared between groups. Multivariate logistic regression analyses were performed to identify factors associated with systemic inflammatory response syndrome (SIRS), sepsis, and polymicrobial infection.

**Results:**

A total of 208 patients with acute biliary infection were included, among whom 61 (29.3%) had polymicrobial infection. Gram-negative bacteria predominated in both groups, with *Escherichia coli* being the most common pathogen. Compared with monomicrobial infection, polymicrobial infection exhibited a relatively higher proportion of Gram-positive organisms. Patients with polymicrobial infection had significantly higher rates of SIRS and sepsis, and polymicrobial infection was independently associated with both SIRS and sepsis. Antibiotic resistance was significantly more common in the polymicrobial group. Among *Escherichia coli* isolates, resistance rates to several commonly used antibiotics were higher in polymicrobial infection, whereas no significant differences were observed for *Klebsiella pneumoniae* or *Enterococcus faecium*. However, antimicrobial resistance itself was not independently associated with SIRS or sepsis. A history of biliary surgery was identified as independent risk factors for polymicrobial infection.

**Conclusions:**

Polymicrobial infection was independently associated with more severe clinical manifestations and a greater antimicrobial resistance burden in acute biliary infection. Prior biliary surgery may help identify patients at increased risk of polymicrobial infection and inform early risk stratification and empirical antimicrobial selection.

## Introduction

Acute biliary infection is a common and potentially life-threatening condition that mainly includes acute cholecystitis and acute cholangitis. It usually develops as a consequence of biliary obstruction with subsequent bacterial colonization (Gomi et al., 2018; Sulzer and Ocuin, 2019). Despite substantial progress in diagnostic approaches and interventional management, acute biliary infection continues to cause considerable morbidity (Lee, 2009; Chen et al., 2024). Therefore, prompt evaluation of disease severity and timely optimization of antimicrobial treatment are crucial for improving patient outcomes.

Although acute cholecystitis and acute cholangitis differ in clinical presentation and underlying mechanisms, they often share overlapping microbial etiologies (Munro and Sorrell, 1986). Acute biliary infections are typically dominated by Gram-negative bacteria, most commonly *Escherichia coli* and *Klebsiella pneumoniae (*Zhang et al., 2024). However, polymicrobial infections are also common and may reflect a more complex infectious process (Vierra et al., 2024). Accumulating evidence suggests that interactions among co-infecting microorganisms can enhance bacterial virulence, modulate host immune responses, and increase disease severity compared with monomicrobial infections (Avadhanula et al., 2006; McCullers, 2014; Li et al., 2015; Tay et al., 2016; Bakaletz, 2017). At the same time, antimicrobial resistance has become an increasing concern in the management of biliary infections (Chen et al., 2021; Cozma et al., 2025). Repeated biliary interventions and extensive antibiotic exposure may disrupt the biliary microbiota and facilitate the emergence of resistant organisms (Gotthardt et al., 2013; Annavajhala et al., 2019; Klein et al., 2022). Polymicrobial infections, in particular, may be associated with microbial communities of greater diversity and higher antimicrobial resistance. Empirical antimicrobial therapy may therefore be less effective (Xiang et al., 2025). Nevertheless, it remains unclear how polymicrobial infection and antimicrobial resistance interact to influence clinical outcomes in acute biliary infection. The risk factors for polymicrobial infection also remain incompletely understood.

Therefore, this study aimed to characterize the clinical impact, antimicrobial resistance profiles, and risk factors of polymicrobial infection in acute biliary infection, with the goal of informing risk stratification and optimizing empirical treatment strategies.

## Materials and methods

### Study design and patient population

This retrospective observational study was conducted at the General Hospital of Ningxia Medical University. During the study period, endoscopic retrograde cholangiopancreatography (ERCP) and percutaneous transhepatic gallbladder drainage (PTGD) were routinely performed as part of the clinical management of eligible patients, with clinical and microbiological information documented in the corresponding hospital records. Consecutive patients diagnosed with acute biliary infection between August 1, 2020 and August 1, 2023 were retrospectively identified from the hospital medical records and screened for eligibility. Acute biliary infection included both acute cholecystitis and acute cholangitis. The study flowchart is shown in **Figure 1**.

**Figure 1:**
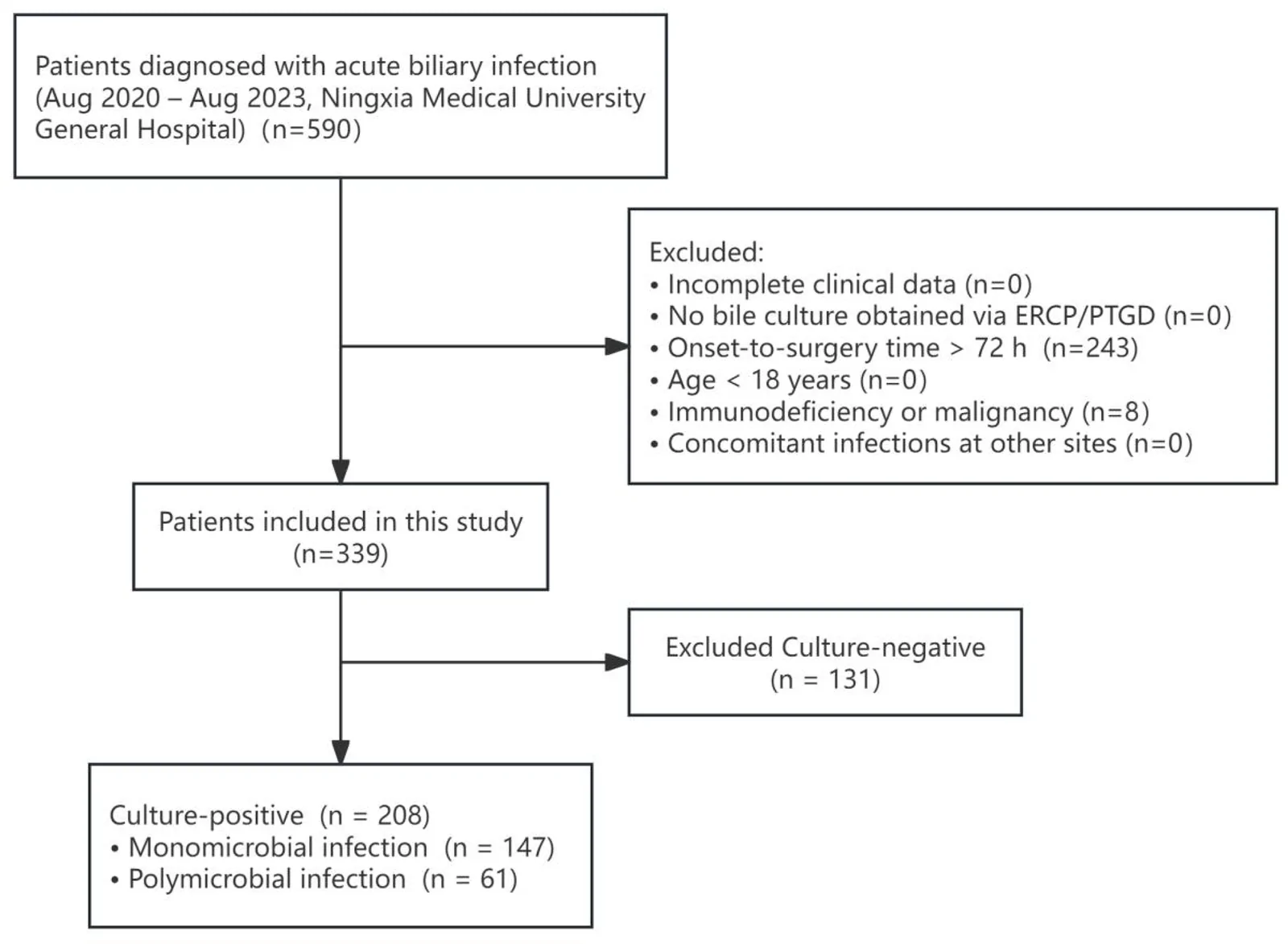
Study flowchart.

Patients were included if they met the following criteria: (1) diagnosis of acute biliary infection, (2) underwent ERCP or PTGD, and (3) had intraoperative bile samples collected for microbiological culture. Patients were excluded if they had (1) incomplete medical records, (2) age younger than 18 years, (3) not undergo ERCP or PTGD or did not have bile culture performed, (4) an interval of more than 72 hours from symptom onset to ERCP or PTGD procedures, (5) concomitant immunodeficiency or malignancy, or (6) concurrent acute or chronic infection at other sites.

Clinical data were retrospectively extracted from electronic medical records using a standardized data collection form. The collected variables included demographic characteristics (age, sex, and body mass index), clinical features (fever, diabetes mellitus, history of biliary surgery, severity level, and bile source), laboratory parameters (white blood cell count, glucose, total bilirubin, and alanine aminotransferase), microbiological characteristics (drug resistance), preoperative antibiotic exposure, morbidity (systemic inflammatory response syndrome(SIRS), sepsis, and organ failure(OF)), and length of hospital stay.

This study was reported in accordance with the Strengthening the Reporting of Observational Studies in Epidemiology (STROBE) guidelines (Cuschieri, 2019). The study was approved by the Institutional Review Board of Ningxia Medical University General Hospital (Ethics ID 2019–466). Because this was a retrospective study based on anonymized clinical data and bile samples collected during routine procedures (ERCP and PTGD) for standard clinical care, the requirement for informed consent was waived by the Ethics Committee.

### Definitions

Patients were categorized into monomicrobial and polymicrobial infection groups based on bile culture results. Monomicrobial infection was defined as the isolation of a single microorganism from bile culture, whereas polymicrobial infection was defined as the isolation of two or more microorganisms. The diagnosis and severity grading of acute cholangitis and acute cholecystitis were based on the Tokyo Guidelines 2018 (TG18). Patients were diagnosed according to the corresponding TG18 diagnostic criteria and classified as having mild, moderate, or severe disease using the respective TG18 severity grading criteria (Kiriyama et al., 2018; Yokoe et al., 2018). SIRS was defined according to standard clinical criteria, including abnormalities in body temperature, heart rate, respiratory rate, and white blood cell count (Baddam and Burns, 2026). Sepsis was defined in accordance with the Sepsis-3 criteria as life-threatening organ dysfunction resulting from a dysregulated host response to infection (Seymour et al., 2016). A history of biliary surgery was defined as any prior biliary intervention, including ERCP and non-ERCP procedures. For the purpose of this study, isolates resistant to at least one tested antimicrobial agent were classified as antimicrobial-resistant. Multidrug-resistant (MDR) organisms were defined according to the international consensus criteria proposed by Magiorakos et al (Magiorakos et al., 2012).

### Sample collection and microbiological analysis

During PTGD or ERCP, all procedure-related instruments were strictly sterilized. Bile samples were collected intraoperatively under aseptic conditions. To minimize contamination, the initial 2 mL of aspirated bile was discarded, and fresh bile was obtained using a sterile disposable syringe. The samples were transferred into sterile collection tubes and promptly sent to the clinical microbiology laboratory for culture. Bile specimens were inoculated into BACT/ALERT culture bottles (bioMérieux, France). The culture bottles were incubated at 35–37 °C in the BACT/ALERT 3D automated microbial detection system (bioMérieux, France) until microbial growth was detected or the incubation period was completed. Positive cultures were subjected to Gram staining and subsequently subcultured onto appropriate solid media to obtain pure isolates. Bacterial identification and antimicrobial susceptibility testing were performed using the VITEK-2 Compact system (bioMérieux, France). Quality control was performed using the reference strains *Enterococcus faecalis* ATCC 29212, *Escherichia coli* ATCC 25922, and *Klebsiella pneumoniae* ATCC 700603. Antimicrobial susceptibility results were interpreted according to the Clinical and Laboratory Standards Institute guidelines.

### Statistical analysis

All statistical analyses were performed using R version 4.3.3. Continuous variables were expressed as mean ± standard deviation (SD) or median (interquartile range, IQR), and categorical variables were presented as counts and percentages. Comparisons between monomicrobial and polymicrobial groups were performed using the Student’s t-test or Mann–Whitney U test for continuous variables, and the chi-square test or Fisher’s exact test for categorical variables. Univariate and multivariate logistic regression analyses were performed to identify factors associated with SIRS, sepsis, and polymicrobial infection. Variables with a P value < 0.20 in univariate analysis were entered into multivariate logistic regression models. Odds ratios (OR) and 95% confidence intervals (CI) were calculated. A two-sided P value < 0.05 was considered statistically significant. Sensitivity analyses were performed by repeating the primary analyses in the expanded cohort without restricting the interval between symptom onset and biliary intervention. Because only one patient had an extensively drug-resistant (XDR) isolate, XDR isolates were combined with the MDR group for subsequent analyses.

## Results

### Baseline characteristics

A total of 208 patients with acute biliary infection were included in this study, of whom 147 (70.7%) had monomicrobial infection and 61 (29.3%) had polymicrobial infection. The baseline characteristics of the two groups are summarized in **Table 1**. Compared with the monomicrobial group, patients with polymicrobial infection were slightly older (median age: 74.00 vs. 72.00 years, *P* = 0.049) and had a shorter time from onset to procedures (28.00 vs. 41.00 hours, *P* = 0.005). Drug resistance was significantly more common in the polymicrobial group than in the monomicrobial group (86.89% vs. 58.50%, *P* < 0.001). In addition, patients with polymicrobial infection had significantly higher rates of SIRS (85.25% vs. 66.67%, *P* = 0.007) and sepsis (83.61% vs. 66.67%, *P* = 0.014). No significant differences were observed between the two groups in terms of sex distribution, body mass index, presence of fever, diabetes mellitus, history of biliary surgery, source of bile, severity level, OF, or length of hospital stay, preoperative antibiotic exposure, white blood cell count, glucose, total bilirubin, or alanine aminotransferase levels (all *P* > 0.05).

**Table 1:** Baseline characteristics stratified by infection type.

Characteristic	Total (n = 208)	Monomicrobial(n = 147)	Polymicrobial(n = 61)	*P*
Age, median (IQR)	73.00 (64.75, 80.00)	72.00 (63.00, 79.00)	74.00 (68.00, 82.00)	**0.049**
Sex, n (%)				0.390
Female	74 (35.58)	55 (37.41)	19 (31.15)	
Male	134 (64.42)	92 (62.59)	42 (68.85)	
BMI, Mean ± SD	24.10 ± 3.84	24.34 ± 3.81	23.51 ± 3.86	0.156
Time to surgery, median (IQR)	38.50 (26.00, 54.00)	41.00 (28.00, 56.00)	28.00 (25.00, 50.00)	**0.005**
Fever, n (%)	80 (38.46)	54 (36.73)	26 (42.62)	0.427
Diabetes mellitus, n (%)	34 (16.35)	26 (17.69)	8 (13.11)	0.417
History of biliary surgery, n (%)	66 (31.73)	41 (27.89)	25 (40.98)	0.065
Source of bile, n (%)				0.964
Gallbladder	55 (26.44)	39 (26.53)	16 (26.23)	
Bile duct	153 (73.56)	108 (73.47)	45 (73.77)	
Severity level, n (%)				0.617
Mild	80 (38.46)	59 (40.14)	21 (34.43)	
Moderate	69 (33.17)	49 (33.33)	20 (32.79)	
Severe	59 (28.37)	39 (26.53)	20 (32.79)	
Drug resistance, n (%)	139 (66.83)	86 (58.50)	53 (86.89)	**<0.001**
Length of hospital stay, median (IQR)	5.00 (3.00, 7.00)	5.00 (3.00, 7.00)	5.00 (3.00, 7.00)	0.749
Preoperative antibiotic exposure, n (%)	205 (98.56)	145 (98.64)	60 (98.36)	1.000
Sepsis, n(%)	149 (71.63)	98 (66.67)	51 (83.61)	**0.014**
SIRS, n(%)	150 (72.12)	98 (66.67)	52 (85.25)	**0.007**
OF, n(%)	38 (18.27)	25 (17.01)	13 (21.31)	0.465
WBC, median (IQR)	11.48 (8.39, 15.56)	11.27 (8.01, 15.73)	11.69 (9.02, 15.24)	0.547
Glucose, median (IQR)	6.58 (5.71, 8.24)	6.58 (5.76, 8.41)	6.58 (5.61, 7.70)	0.411
TBil, median (IQR)	67.80 (28.62, 103.55)	62.00 (27.25, 98.60)	75.20 (33.50, 112.90)	0.155
ALT, median (IQR)	157.05 (50.90, 281.10)	151.40 (48.85, 268.00)	173.60 (75.40, 303.10)	0.240

Bold values indicate statistical significance (P < 0.05).

SD, standard deviation; IQR, interquartile range; BMI, body mass index; SIRS, systemic inflammatory response syndrome; OF, organ failure; WBC, white blood cell; TBil, total bilirubin; ALT, alanine aminotransferase.

### Microbial profile

The microbial profile of biliary infection is presented in **Figure 2**. A total of 279 bacterial isolates were recovered from bile cultures, including 147 isolates from the monomicrobial infection group and 132 isolates from the polymicrobial infection group. Most isolates were Gram-negative bacteria, followed by Gram-positive bacteria, whereas fungal isolates were uncommon (one isolate in each group) (**Figure 2A**). Compared with the monomicrobial group, the polymicrobial group showed a relatively higher proportion of Gram-positive organisms. At the species level, *Escherichia coli* was the most frequently isolated pathogen in both the monomicrobial and polymicrobial infection groups (71/147, 48.3% vs. 50/132, 37.9%), followed by *Klebsiella pneumoniae* (21/147, 14.3% vs. 11/132, 8.3%) and *Enterococcus faecium* (11/147, 7.5% vs. 11/132, 8.3%) (**Figure 2B**). Other organisms were identified less frequently. These findings indicated that Gram-negative bacteria, particularly *Escherichia coli*, were the predominant pathogens in acute biliary infection.

**Figure 2:**
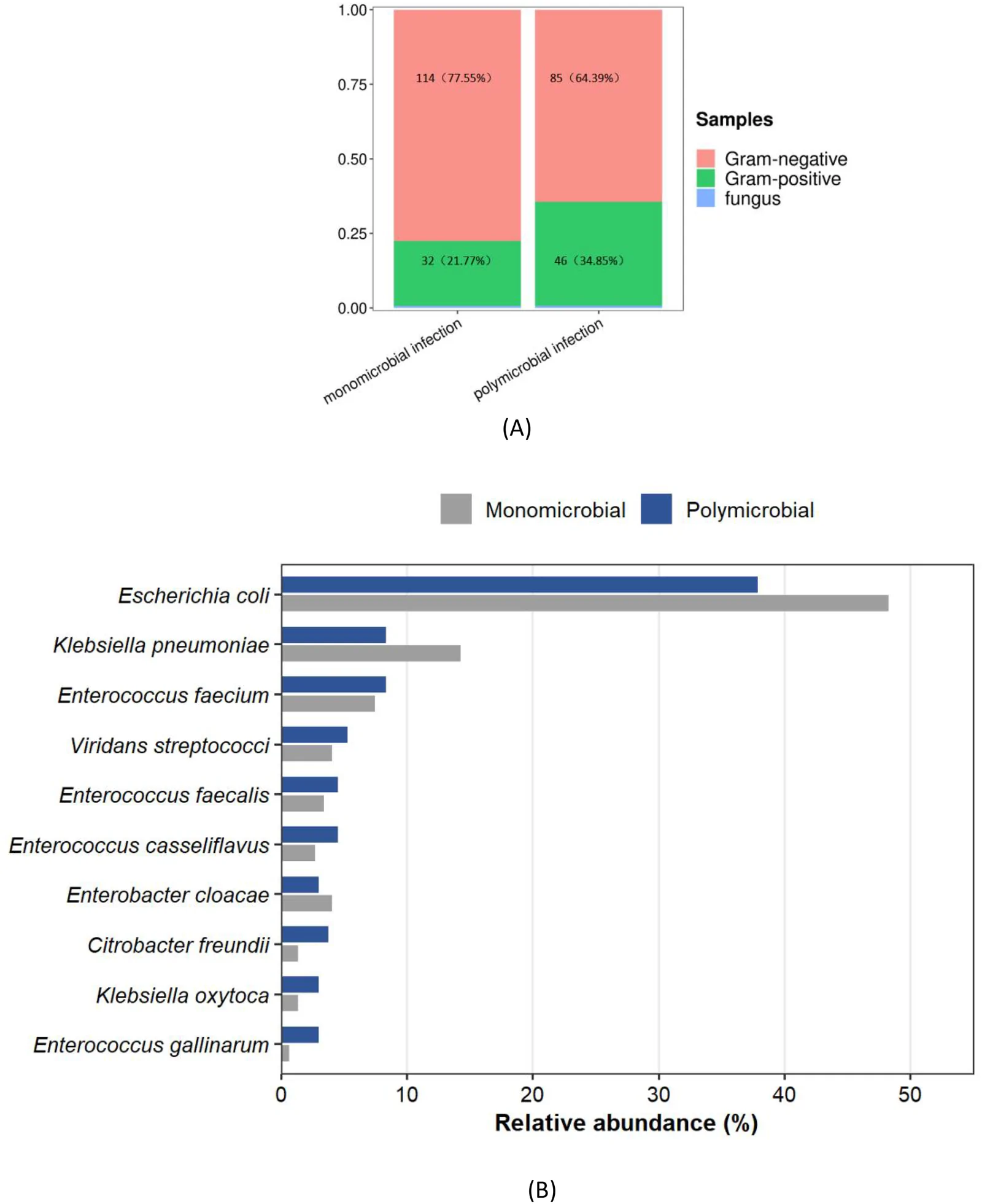
Microbial profile in monomicrobial and polymicrobial infections. **(A)** Microbial composition. **(B)** Predominant bacterial species.

### Antibiotic resistance profiles

To evaluate the association between antimicrobial resistance and clinical outcomes, the antimicrobial resistance profiles of the three most frequently isolated pathogens were first compared between the monomicrobial and polymicrobial infection groups (**Figure 3**), followed by analyses of the associations between antimicrobial resistance and clinical outcomes (**Table 2**). Among *Escherichia coli* isolates, resistance rates to ciprofloxacin, ampicillin, and piperacillin were significantly higher in the polymicrobial group than in the monomicrobial group (all *P* < 0.05), with the highest resistance observed for ampicillin (74%). In contrast, the antimicrobial resistance patterns of *Klebsiella pneumoniae* were generally comparable between the two groups, with overall resistance rates remaining relatively low (all < 40%). Similarly, resistance profiles of *Enterococcus faecium* were broadly similar between groups, although consistently high resistance to erythromycin was observed, reaching 90.91% in the polymicrobial group. No statistically significant differences were identified for *Klebsiella pneumoniae* or *Enterococcus faecium* between the two groups (*P* > 0.05). Additional analyses showed that antimicrobial resistance itself was not significantly associated with SIRS, sepsis, OF, or length of hospital stay (**Table 2**). Similar findings were observed after further categorizing resistant isolates into resistant but non-MDR and MDR groups (**Supplementary Table S5**). Likewise, pathogen-specific analyses showed that antimicrobial resistance in *Escherichia coli, Klebsiella pneumoniae*, and *Enterococcus faecium* was not independently associated with adverse clinical outcomes (**Supplementary Tables S6**–**S8**).

**Figure 3:**
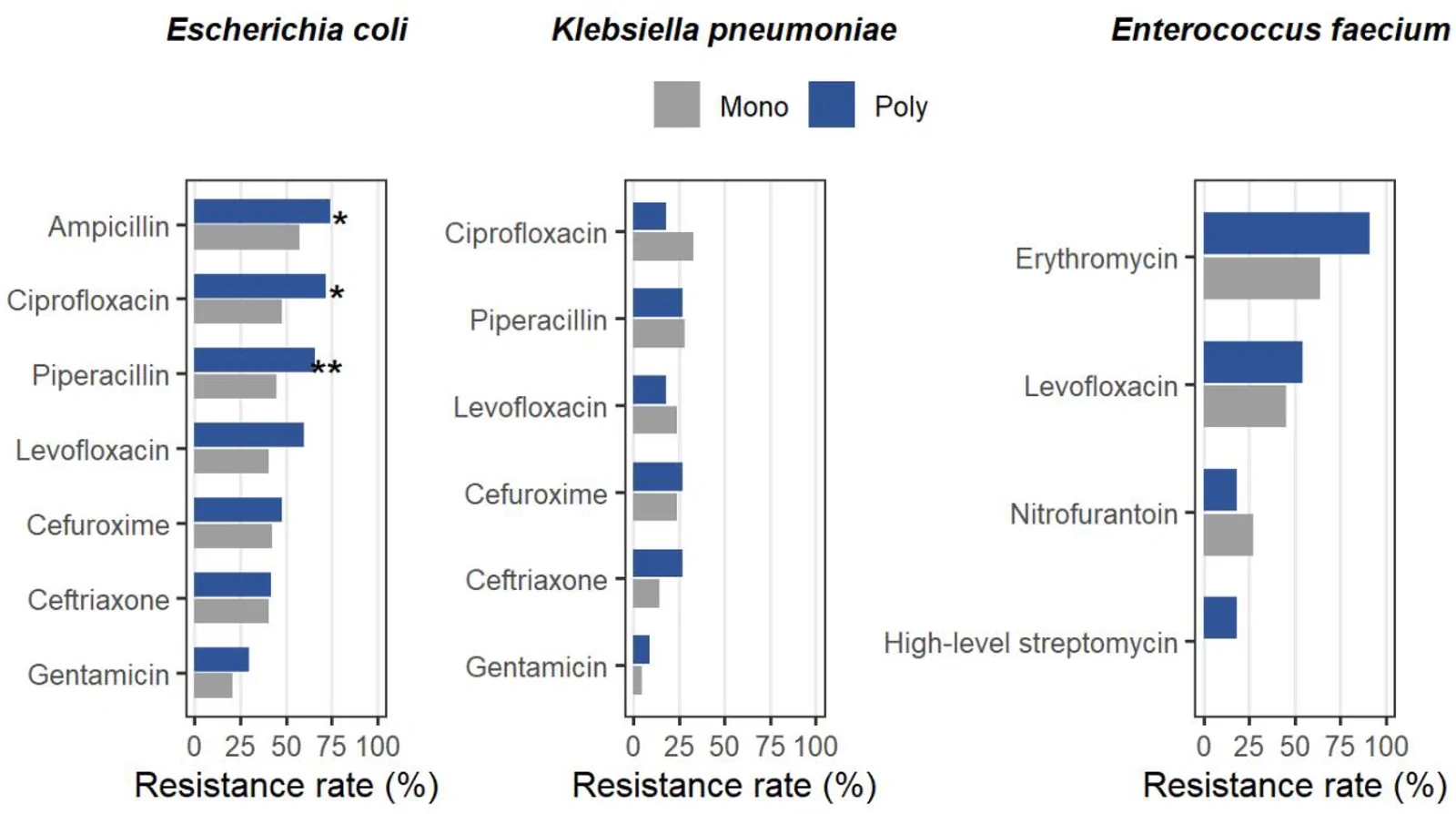
Comparison of antibiotic resistance profiles between monomicrobial and polymicrobial infections. *P < 0.05; **P < 0.01.

**Table 2:** Comparison of clinical outcomes according to antimicrobial resistance status.

Variables	Total (n = 208)	Drug resistance (n = 139)	Susceptible (n = 69)	*P*
Length of hospital stay, median (IQR)	5.00 (3.00, 7.00)	5.00 (3.00, 6.50)	5.00 (3.00, 8.00)	0.829
Sepsis, n(%)	149 (71.63)	102 (73.38)	47 (68.12)	0.428
SIRS, n(%)	150 (72.12)	102 (73.38)	48 (69.57)	0.563
OF, n(%)	38 (18.27)	23 (16.55)	15 (21.74)	0.362

Bold values indicate statistical significance (P < 0.05).

### Logistic regression analysis for clinical outcomes

Multivariate logistic regression analyses were performed to identify independent factors associated with SIRS and sepsis (**Tables 3**, **4**). After adjustment for potential confounders, polymicrobial infection was identified as an independent risk factor for both SIRS (OR = 2.84, 95% CI: 1.20 - 6.74, *P* = 0.018) and sepsis (OR = 2.33, 95% CI: 1.01 - 5.40, *P* = 0.048), whereas antimicrobial resistance was not independently associated with either outcome.

**Table 3:** Logistic regression analysis of factors associated with SIRS.

Variables	Univariate analysis		Multivariate analysis	
	OR (95%CI)	*P*	OR (95%CI)	*P*
Age	1.01 (0.99 ~ 1.04)	0.311		
Sex				
Female	1.00 (Reference)		1.00 (Reference)	
Male	1.73 (0.93 ~ 3.21)	0.085	1.53 (0.74 ~ 3.18)	0.253
BMI	1.01 (0.93 ~ 1.09)	0.852		
Diabetes mellitus				
No	1.00 (Reference)			
Yes	0.91 (0.41 ~ 2.05)	0.828		
History of biliary surgery				
No	1.00 (Reference)		1.00 (Reference)	
Yes	0.61 (0.32 ~ 1.15)	0.128	0.62 (0.31 ~ 1.26)	0.188
Drug resistance				
No	1.00 (Reference)			
Yes	1.21 (0.64 ~ 2.28)	0.564		
Preoperative antibiotic exposure				
No	1.00 (Reference)		1.00 (Reference)	
Yes	5.32 (0.47 ~ 59.85)	0.176	5.82 (0.39 ~ 86.99)	0.201
Source of bile				
Gallbladder	1.00 (Reference)			
Bile duct	0.85 (0.42 ~ 1.70)	0.640		
Infection type				
Monomicrobial	1.00 (Reference)		1.00 (Reference)	
Polymicrobial	2.89 (1.32 ~ 6.34)	0.008	2.84 (1.20 ~ 6.74)	**0.018**
Time to surgery	0.99 (0.97 ~ 1.00)	0.144	0.99 (0.97 ~ 1.01)	0.393
TBIL	1.01 (1.01 ~ 1.01)	0.038	1.01 (1.00 ~ 1.01)	0.164

Bold values indicate statistical significance (P < 0.05).

OR, Odds ratios, CI, confidence intervals.

**Table 4:** Logistic regression analysis of factors associated with sepsis.

Variables	Univariate analysis		Multivariate analysis	
	OR (95%CI)	*P*	OR (95%CI)	*P*
Age	1.02 (0.99 ~ 1.04)	0.133	1.02 (0.99 ~ 1.05)	0.140
Sex				
Female	1.00 (Reference)		1.00 (Reference)	
Male	2.02 (1.09 ~ 3.75)	0.026	1.89 (0.92 ~ 3.92)	0.085
BMI	1.01 (0.93 ~ 1.09)	0.845		
Diabetes mellitus				
No	1.00 (Reference)			
Yes	0.94 (0.42 ~ 2.11)	0.882		
History of biliary surgery				
No	1.00 (Reference)		1.00 (Reference)	
Yes	0.63 (0.34 ~ 1.19)	0.159	0.64 (0.32 ~ 1.31)	0.226
Drug resistance				
No	1.00 (Reference)			
Yes	1.29 (0.69 ~ 2.42)	0.428		
Preoperative antibiotic exposure				
No	1.00 (Reference)		1.00 (Reference)	
Yes	5.19 (0.46 ~ 58.39)	0.182	6.28 (0.41 ~ 96.60)	0.188
Source of bile				
Gallbladder	1.00 (Reference)			
Bile duct	0.93 (0.47 ~ 1.85)	0.834		
Infection type				
Monomicrobial	1.00 (Reference)		1.00 (Reference)	
Polymicrobial	2.55 (1.19 ~ 5.45)	0.016	2.33 (1.01 ~ 5.40)	**0.048**
Time to surgery	0.99 (0.97 ~ 1.00)	0.119	0.99 (0.97 ~ 1.01)	0.324
TBIL	1.01 (1.01 ~ 1.01)	0.031	1.01 (1.00 ~ 1.01)	0.200

Bold values indicate statistical significance (P < 0.05).

### Risk factors for polymicrobial infection

Multivariate logistic regression analysis was performed to identify risk factors for polymicrobial infection (**Table 5**). A history of biliary surgery (OR = 2.34, 95% CI: 1.15 - 4.74, *P* = 0.018) and a shorter interval from onset to procedures (OR = 0.98, 95% CI: 0.96 - 0.99, *P* = 0.011) were identified as independent risk factors for polymicrobial infection.To further explore the impact of different types of prior biliary procedures, subgroup analysis was conducted among patients with a history of biliary surgery (**Table 6**). Although the proportion of polymicrobial infection was higher in patients who had undergone ERCP compared with those who had undergone non-ERCP procedures, the difference was not statistically significant. These findings suggest that prior biliary interventions may contribute to the development of polymicrobial infection, while the specific role of ERCP requires further investigation.

**Table 5:** Multivariate logistic regression analysis of risk factors for polymicrobial infection.

Variables	Univariate analysis		Multivariate analysis	
	OR (95%CI)	*P*	OR (95%CI)	*P*
Age	1.02 (1.00 ~ 1.05)	0.077	1.03 (1.00 ~ 1.06)	0.079
Sex				
Female	1.00 (Reference)			
Male	1.32 (0.70 ~ 2.50)	0.391		
BMI	0.94 (0.87 ~ 1.02)	0.157	0.96 (0.87 ~ 1.05)	0.330
Diabetes mellitus				
No	1.00 (Reference)			
Yes	0.70 (0.30 ~ 1.65)	0.419		
History of biliary surgery				
No	1.00 (Reference)		1.00 (Reference)	
Yes	1.80 (0.96 ~ 3.35)	0.066	2.34 (1.15 ~ 4.74)	**0.018**
Preoperative antibiotic exposure				
No	1.00 (Reference)			
Yes	0.83 (0.07 ~ 9.30)	0.878		
Source of bile				
Gallbladder	1.00 (Reference)			
Bile duct	1.02 (0.52 ~ 2.00)	0.964		
Fever				
No	1.00 (Reference)			
Yes	1.28 (0.70 ~ 2.35)	0.427		
Time to surgery	0.97 (0.96 ~ 0.99)	0.005	0.98 (0.96 ~ 0.99)	**0.011**
WBC	1.01 (0.96 ~ 1.07)	0.659		
TBIL	1.00 (1.00 ~ 1.01)	0.106	1.01 (1.00 ~ 1.01)	0.058
ALT	1.00 (1.00 ~ 1.00)	0.074	1.00 (1.00 ~ 1.00)	0.170

Bold values indicate statistical significance (P < 0.05).

**Table 6:** Association between ERCP and polymicrobial infection among patients with prior biliary surgery.

Variables	Total (n = 66)	Non-ERCP (n = 26)	ERCP (n = 40)	Statistic	*P*
Infection type, n(%)				χ²=2.19	0.139
Monomicrobial	41 (62.12)	19 (73.08)	22 (55.00)		
Polymicrobial	25 (37.88)	7 (26.92)	18 (45.00)		

χ², Chi-square test; ERCP, endoscopic retrograde cholangiopancreatography.

To evaluate the impact of the 72-hour inclusion criterion, sensitivity analyses were performed in the full cohort without restricting the interval between symptom onset and biliary intervention. The baseline characteristics and multivariable analyses remained generally consistent with those of the primary analyses (**Supplementary Tables S1**–**S4**).

## Discussion

In this single-center retrospective cohort study, we investigated the clinical characteristics, microbiological profiles, and antimicrobial resistance patterns of 208 patients with acute biliary infection who underwent ERCP or PTGD within 72 hours of symptom onset, with a particular focus on the clinical significance of polymicrobial infection. We found that polymicrobial infection was relatively common, accounting for 29.3% of all cases. Microbiological analysis demonstrated that Gram-negative bacteria remained the predominant pathogens, with *Escherichia coli* being the most frequently isolated organism. Compared with monomicrobial infection, polymicrobial infection was characterized by a higher proportion of Gram-positive organisms. Antimicrobial susceptibility testing further showed that *Escherichia coli* isolates from the polymicrobial group exhibited higher resistance rates to several commonly used antimicrobial agents. However, antimicrobial resistance itself was not independently associated with adverse clinical outcomes. Multivariable analysis further demonstrated that polymicrobial infection was independently associated with both SIRS and sepsis, whereas previous biliary surgery and a shorter interval from symptom onset to biliary intervention were independently associated with polymicrobial infection.

In the present study, polymicrobial infection was relatively common, accounting for 29.3% of patients with acute biliary infection. Previous studies have consistently shown that polymicrobial infections are a relatively common type of infection in acute biliary tract infections, but there are certain differences in the reported incidence rates among different studies. Shenoy et al. reported polymicrobial infection in 31.5% of culture-positive bile samples from patients with cholangitis in India (Shenoy et al., 2014), while Lee et al. reported a polymicrobial infection rate of 26.9% in patients with bacteremic biliary tract infection in Taiwan (Lee et al., 2007), and an Australian population-based study reported polymicrobial infection in 18.4% of cholangitis-associated bloodstream infections (Gassiep et al., 2024). In contrast, a Korean study based on bile cultures found multiple organisms in 39.1% of specimens (Goo et al., 2012). The prevalence observed in the present study (29.3%) falls within the range reported in the literature, and the variability among studies is likely attributable not only to geographical differences but also to differences in study populations, specimen types, and microbiological sampling strategies. In addition, patients with polymicrobial infection were significantly older than those with monomicrobial infection. Although age was not independently associated with polymicrobial infection in the multivariable analysis, older patients generally have more complicated biliary disease, higher rates of biliary bacterial colonization, and greater healthcare exposure, all of which may increase the opportunity for polymicrobial infection (Stewart et al., 2008).

Microbiological analysis demonstrated that Gram-negative bacteria remained the predominant pathogens in acute biliary infection, with *Escherichia coli* being the most frequently isolated organism, followed by *Klebsiella pneumoniae*. These findings are in line with previous reports identifying Gram-negative organisms as the principal pathogens in biliary infections (Wu et al., 2021; Chen et al., 2024). Compared with monomicrobial infection, polymicrobial infection in our cohort was characterized by a higher proportion of Gram-positive organisms, among which *Enterococcus faecium* remained the predominant Gram-positive isolate. Similar microbiological patterns have been reported previously, particularly in patients with a history of ERCP, biliary stent placement, or prior biliary surgery, in whom *Enterococcus* species are isolated more frequently (Lorenz et al., 1998; Gargouri et al., 2015; Mussa et al., 2022). Previous studies have suggested that biliary interventions may alter the normal biliary environment and facilitate the retrograde colonization of enteric microorganisms (Li et al., 2026). In addition, repeated hospitalization and prior exposure to broad-spectrum antimicrobial agents may further disrupt the biliary microbiota, favoring the persistent colonization of relatively resilient organisms such as *Enterococcus* spp (Ramirez et al., 2020). Furthermore, prolonged biliary obstruction, bile stasis, and biofilm formation may promote the coexistence of multiple bacterial species within the biliary tract, thereby contributing to a more complex microbial community (Sung et al., 1993; Carpenter, 1998). Nevertheless, despite the increased proportion of Gram-positive organisms, *Escherichia coli* remained the predominant pathogen in polymicrobial infection. This finding suggests that polymicrobial infection is characterized by an expansion of the microbial spectrum rather than a replacement of the dominant pathogen, with Gram-positive organisms coexisting alongside enteric Gram-negative bacteria.

In addition to differences in microbial composition, we found that polymicrobial infection was associated with a significantly higher prevalence of antimicrobial-resistant isolates than monomicrobial infection, indicating a greater antimicrobial resistance burden in patients with polymicrobial infection. Previous studies have similarly reported a higher prevalence of antimicrobial-resistant pathogens among patients with polymicrobial infections, while prior biliary interventions, repeated hospitalization, and previous antimicrobial exposure have all been recognized as important risk factors for colonization or infection with resistant organisms in biliary tract infections (Mussa et al., 2022; Chen et al., 2024; Stathopoulos et al., 2024), consistent with recent evidence highlighting the association of healthcare exposure and prior biliary interventions with polymicrobial and multidrug-resistant biliary infections (Toma et al., 2025). Further analysis of the three predominant pathogens showed that only *Escherichia coli* isolates from the polymicrobial group exhibited significantly higher resistance rates to ciprofloxacin, ampicillin, and piperacillin, whereas the resistance profiles of *Klebsiella pneumoniae* and *Enterococcus faecium* were generally comparable between the two groups. These findings suggest that the increased antimicrobial resistance burden associated with polymicrobial infection is primarily attributable to *Escherichia coli*, rather than representing a generalized increase in resistance across all predominant pathogens. Previous studies have likewise shown that *Escherichia coli* is not only the most common pathogen causing acute biliary infection but also the principal source of extended spectrum β-lactamase (ESBL) producing and MDR isolates (Alipour et al., 2024), making its resistance profile particularly relevant when selecting empirical antimicrobial therapy.

Notably, despite the higher overall prevalence of antimicrobial resistance in the polymicrobial group, antimicrobial resistance was not independently associated with SIRS, sepsis, organ failure, or length of hospital stay in our study. Similar findings were observed after incorporating MDR classification into the analysis. These results suggest that antimicrobial resistance alone may not directly determine short-term clinical outcomes in patients with acute biliary infection (Nve et al., 2023). The Tokyo Guidelines 2018 and other studies have consistently emphasized that adequate source control, particularly timely biliary drainage, is the cornerstone of successful management for acute biliary infection (Kiriyama et al., 2018; Lu et al., 2022; Nve et al., 2023; Cozma et al., 2024). In addition, subsequent adjustment of antimicrobial therapy according to culture and susceptibility testing results may further mitigate the adverse impact of antimicrobial resistance on patient outcomes (Gomi et al., 2018; Tamma et al., 2024). Therefore, although antimicrobial resistance increases the complexity of empirical antimicrobial therapy in patients with polymicrobial infection, it does not appear to fully explain the higher incidences of SIRS and sepsis observed in this population.

Multivariable analysis further demonstrated that polymicrobial infection was independently associated with both SIRS and sepsis, suggesting that polymicrobial infection is not merely a microbiological finding but may also serve as a marker of a more complex biliary infectious state. This observation is consistent with previous studies of acute biliary infection and other complicated intra-abdominal infections, which have similarly reported that polymicrobial infection is associated with an increased risk of systemic inflammatory complications, sepsis, and adverse clinical outcomes (Napolitano, 2022; Bongers et al., 2024; Smith, 2024). Notably, previous biliary surgery was independently associated with polymicrobial infection in our cohort. Taken together with previous studies, this finding suggests that polymicrobial infection is more likely to occur in patients with more complex underlying biliary disease rather than simply reflecting the presence of multiple microorganisms (Tchernin et al., 2022). Prior biliary surgery, repeated biliary interventions, and chronic biliary disorders may alter the biliary anatomy and local microenvironment, thereby facilitating persistent bacterial colonization and the coexistence of multiple pathogens (Shen et al., 2020). In addition, we found that a shorter interval from symptom onset to ERCP/PTGD was independently associated with polymicrobial infection. A more plausible explanation is that patients with more severe disease or rapidly progressive biliary sepsis are more likely to undergo urgent biliary drainage shortly after presentation, rather than early biliary drainage itself promoting polymicrobial infection (Weissman et al., 2023). Collectively, these findings suggest that polymicrobial infection should be regarded as an important clinical marker of increased infection complexity and a higher risk of systemic inflammatory complications. Early recognition of this high-risk infection phenotype may facilitate timely risk stratification and closer clinical monitoring.

This study has several strengths. First, the use of bile cultures obtained during ERCP or PTGD allowed us to define polymicrobial infection based on direct microbiological evidence and to examine its associations with clinical outcomes in patients with acute biliary infection. Second, we evaluated the antimicrobial resistance burden at both the overall level and for the three predominant pathogens, allowing us to determine whether the observed difference in resistance was consistent across major pathogens. Several limitations should also be acknowledged. First, this was a single-center retrospective study, which may limit the generalizability of the findings and introduce potential selection bias. Second, the sample size was relatively small, which may reduce the statistical power to detect certain associations. Third, antimicrobial resistance was evaluated at the organism level, and more detailed characterization of resistance mechanisms was not performed. Fourth, although several independent clinical factors associated with polymicrobial infection were identified, these variables were not integrated into a simplified bedside risk prediction model or externally validated, limiting their immediate clinical applicability. Future multicenter prospective studies are warranted to develop and validate practical risk stratification tools for the early identification of patients at high risk of polymicrobial infection and to determine the optimal management strategies for these patients.

In conclusion, polymicrobial infection was associated with more severe clinical manifestations and a greater antimicrobial resistance burden in patients with acute biliary infection, although antimicrobial resistance itself was not independently associated with adverse clinical outcomes. Prior biliary interventions may help identify patients at increased risk of polymicrobial infection at the initial clinical assessment. These findings highlight the importance of early risk recognition, bile culture, and consideration of local resistance patterns in guiding empirical and subsequent culture-directed antimicrobial therapy. Prospective multicenter studies are needed to validate these findings and establish their clinical utility.

## Data Availability

The raw data supporting the conclusions of this article will be made available by the authors, without undue reservation.
