# Supplementary material for: Polymicrobial infection in acute biliary infection: clinical impact, resistance, and risk factors
Source: Front Cell Infect Microbiol. 2026 Oct 23;16:1893457. doi: 10.3389/fcimb.2026.1893457 (PMC13739527; doi:10.3389/fcimb.2026.1893457)
Supplement: Supplementary file 1 [file DataSheet1.pdf]

Supplementary Table S1. Baseline characteristics of the expanded cohort.

Characteristic	Total (n = 340)	Monomicrobial(n = 245)	Polymicrobial(n = 95)	*P*
Age, median (IQR)	72.00 (63.00, 79.25)	71.00 (61.00, 79.00)	74.00 (67.00, 80.50)	**0.015**
Sex, n (%)				0.262
Female	127 (37.35)	96 (39.18)	31 (32.63)	
Male	213 (62.65)	149 (60.82)	64 (67.37)	
BMI, Mean ± SD	24.22 ± 3.65	24.32 ± 3.58	23.96 ± 3.84	0.421
Time to surgery, median (IQR)	60.50 (34.00, 83.12)	65.00 (37.00, 84.00)	52.00 (26.00, 79.00)	**0.033**
Fever, n (%)	112 (32.94)	76 (31.02)	36 (37.89)	0.226
Diabetes mellitus, n (%)	62 (18.24)	51 (20.82)	11 (11.58)	**0.048**
History of biliary surgery, n (%)	106 (31.18)	69 (28.16)	37 (38.95)	0.054
Source of bile, n (%)				0.687
Gallbladder	93	69（28.16%）	24（25.26%）	
Bile duct	247	176（71.84%）	71（74.74%）	
Severity level, n (%)				0.376
Mild	131 (38.53)	100 (40.82)	31 (32.63)	
Moderate	119 (35.00)	83 (33.88)	36 (37.89)	
Severe	90 (26.47)	62 (25.31)	28 (29.47)	
Drug resistance, n (%)	226 (66.47)	143 (58.37)	83 (87.37)	**<0.001**
Length of hospital stay, median (IQR)	5.00 (3.00, 7.00)	5.00 (3.00, 7.00)	5.00 (4.00, 7.00)	0.564
Preoperative antibiotic exposure, n (%)	329 (96.76)	236 (96.33)	93 (97.89)	0.695
Sepsis, n(%)	222 (65.29)	149 (60.82)	73 (76.84)	**0.005**
SIRS, n(%)	223 (65.59)	149 (60.82)	74 (77.89)	**0.003**
OF, n(%)	58 (17.06)	40 (16.33)	18 (18.95)	0.564
WBC, median (IQR)	10.81 (7.33, 14.74)	10.67 (7.07, 14.93)	11.11 (8.14, 14.38)	0.441
Glucose, median (IQR)	6.55 (5.59, 8.25)	6.55 (5.58, 8.34)	6.58 (5.61, 7.86)	0.645
TBil, median (IQR)	60.10 (24.65, 97.00)	53.80 (23.40, 94.70)	67.70 (25.85, 105.95)	0.142
ALT, median (IQR)	124.50 (44.43, 243.25)	117.00 (44.10, 242.55)	150.80 (46.40, 235.85)	0.436

Supplementary Table S2. Multivariable logistic regression analysis for factors associated with SIRS in the expanded cohort.

Variables	Univariate analysis		Multivariate analysis	
	OR (95%CI)	*P*	OR (95%CI)	*P*
Age	1.02 (1.00-1.04)	0.052	1.02 (1.00-1.04)	0.125
Sex				
Female	1.00 (Reference)			
Male	1.27 (0.80-2.01)	0.311		
BMI	0.99 (0.93-1.05)	0.768		
Diabetes mellitus				
No	1.00 (Reference)			
Yes	1.13 (0.63-2.02)	0.693		
History of biliary surgery				
No	1.00 (Reference)		1.00 (Reference)	
Yes	0.54 (0.33-0.86)	0.01	0.50 (0.30-0.85)	0.010
Drug resistance				
No	1.00 (Reference)			
Yes	1.17 (0.73-1.88)	0.503		
Preoperative antibiotic exposure				
No	1.00 (Reference)		1.00 (Reference)	
Yes	20.75 (2.62-164.18)	0.004	14.48 (1.73-121.25)	0.014
Source of bile				
Gallbladder	1.00 (Reference)			
Bile duct	0.71 (0.43-1.20)	0.201		
Infection type				
Monomicrobial	1.00 (Reference)		1.00 (Reference)	
Polymicrobial	2.27 (1.31-3.93)	0.003	2.22 (1.22-4.02)	**0.009**
Time to surgery	0.99 (0.98-0.99)	<0.001	0.99 (0.98-1.00)	0.003
TBIL	1.01 (1.00-1.01)	0.002	1.01 (1.00-1.01)	0.017

Supplementary Table S3. Multivariable logistic regression analysis for factors associated with sepsis in the expanded cohort.

Variables	Univariate analysis		Multivariate analysis	
	OR (95%CI)	*P*	OR (95%CI)	*P*
Age	1.02 (1.00-1.04)	0.022	1.02 (1.00-1.04)	0.043
Sex				
Female	1.00 (Reference)		1.00 (Reference)	
Male	1.39 (0.88-2.19)	0.164	1.21 (0.72-2.02)	0.466
BMI	0.99 (0.93-1.05)	0.776		
Diabetes mellitus				
No	1.00 (Reference)			
Yes	1.14 (0.64-2.06)	0.654		
History of biliary surgery				
No	1.00 (Reference)		1.00 (Reference)	
Yes	0.55 (0.34-0.88)	0.013	0.52 (0.31-0.87)	0.013
Drug resistance				
No	1.00 (Reference)			
Yes	1.22 (0.76-1.95)	0.407		
Preoperative antibiotic exposure				
No	1.00 (Reference)		1.00 (Reference)	
Yes	20.46 (2.59-161.92)	0.004	14.24 (1.69-120.02)	0.015
Source of bile				
Gallbladder	1.00 (Reference)			
Bile duct	0.75 (0.45-1.26)	0.275		
Infection type				
Monomicrobial	1.00 (Reference)		1.00 (Reference)	
Polymicrobial	2.14 (1.24-3.67)	0.006	2.01 (1.12-3.63)	**0.020**
Time to surgery	0.99 (0.98-0.99)	<0.001	0.99 (0.98-1.00)	0.003
TBIL	1.01 (1.00-1.01)	0.001	1.01 (1.00-1.01)	0.023

Supplementary Table S4. Multivariable logistic regression analysis for factors associated with polymicrobial infection in the expanded cohort.

Variables	Univariate analysis		Multivariate analysis	
	OR (95%CI)	*P*	OR (95%CI)	*P*
Age	1.02 (1.00-1.05)	0.025	1.03 (1.00-1.05)	0.026
Sex				
Female	1.00 (Reference)			
Male	1.33 (0.81-2.19)	0.263		
BMI	0.97 (0.91-1.04)	0.42		
Diabetes mellitus				
No	1.00 (Reference)		1.00 (Reference)	
Yes	0.50 (0.25-1.00)	0.051	0.50 (0.25-1.03)	0.059
History of biliary surgery				
No	1.00 (Reference)		1.00 (Reference)	
Yes	1.63 (0.99-2.68)	0.055	1.71 (1.02-2.85)	**0.041**
Preoperative antibiotic exposure				
No	1.00 (Reference)			
Yes	1.77 (0.38-8.36)	0.469		
Source of bile				
Gallbladder	1.00 (Reference)			
Bile duct	1.16 (0.68-1.99)	0.591		
Fever				
No	1.00 (Reference)			
Yes	1.36 (0.83-2.23)	0.227		
Time to surgery	0.99 (0.98-1.00)	0.032	0.99 (0.98-1.00)	0.057
WBC	1.01 (0.97-1.06)	0.532		
TBIL	1.00 (1.00-1.01)	0.118	1.00 (1.00-1.01)	0.102
ALT	1.00 (1.00-1.00)	0.258		

Supplementary Table S5. Association of antimicrobial susceptibility status with adverse clinical Outcomes.

Variables	Total (n = 208)	Susceptible (n=69)	Resistant but non-MDR (n =38)	MDR (n =101)	*P*
Length of hospital stay, median (IQR)	5.00 (3.00, 7.00)	5.00 (3.00,8.00)	5.00 (4.00,7.00)	5.00 (3.00,6.00)	0.497
Sepsis, n(%)	149 (71.63)	47 (68.12)	29 (76.32)	73 (72.28)	0.654
SIRS, n(%)	150 (72.12)	48 (69.57)	28 (73.68)	74 (73.27)	0.845
OF, n(%)	38 (18.27)	15 (21.74)	5 (13.16)	18 (17.82)	0.539

Supplementary Table S6. Association between antimicrobial resistance of *Escherichia coli* and adverse clinical outcomes.

Variables	Total (n = 117)	Drug resistance (n = 89)	Susceptible (n = 28)	*P*
Length of hospital stay, median (IQR)	5.00 (3.00, 7.00)	5.00 (3.00, 7.00)	4.00 (3.00, 7.00)	0.300
Sepsis, n(%)	86 (73.50)	66 (74.16)	20 (71.43)	0.968
SIRS, n(%)	87 (74.36)	67 (75.28)	20 (71.43)	0.874
OF, n(%)	28 (23.93)	18 (20.22)	10 (35.71)	0.155

Supplementary Table S7. Association between antimicrobial resistance of *Klebsiella pneumoniae* and adverse clinical outcomes.

Variables	Total (n = 32)	Drug resistance (n = 14)	Susceptible (n = 18)	*P*
Length of hospital stay, median (IQR)	6.00 (3.00, 8.00)	5.50 (3.25, 6.75)	6.00 (3.50, 8.00)	0.454
Sepsis, n(%)	24 (75.00)	11 (78.57)	13 (72.22)	1.000
SIRS, n(%)	24 (75.00)	10 (71.43)	14 (77.78)	1.000
OF, n(%)	6 (18.75)	3 (21.43)	3 (16.67)	1.000

Supplementary Table S8. Association between antimicrobial resistance of *Enterococcus faecium* and adverse clinical outcomes.

Variables	Total (n = 22)	Drug resistance (n = 18)	Susceptible (n = 4)	*P*
Length of hospital stay, median (IQR)	5.00 (3.25, 9.50)	5.00 (4.00, 9.50)	3.00 (2.75, 5.00)	0.184
Sepsis, n(%)	16 (72.73)	14 (77.78)	2 (50.00)	0.292
SIRS, n(%)	16 (72.73)	14 (77.78)	2 (50.00)	0.292
OF, n(%)	5 (22.73)	5 (27.78)	0 (0.00)	0.535
